# Not All Locations Are Created Equal: Exploring How Adults Hide and Search for Objects

**DOI:** 10.1371/journal.pone.0036993

**Published:** 2012-05-11

**Authors:** Eric L. G. Legge, Marcia L. Spetch, Andrew Cenkner, Vadim Bulitko, Craig Anderson, Matthew Brown, Donald Heth

**Affiliations:** 1 Department of Psychology, University of Alberta, Edmonton, Alberta, Canada; 2 Department of Computing Science, University of Alberta, Edmonton, Alberta, Canada; 3 Department of Psychiatry, University of Alberta, Edmonton, Alberta, Canada; University of Ulster, United Kingdom

## Abstract

Little is known about the strategies people use to effectively hide objects from others, or to search for objects others have hidden. The present research extends a recent investigation of people’s hiding and searching strategies in a simple room with 9 cache location. In the present studies, people hid and searched for three objects under more than 70 floor tiles in complex real and virtual rooms. Experiment 1 replicated several finding of Talbot et al within the more complex real and virtual environments. Specifically, people traveled further from origin and selected more dispersed locations when hiding than when searching. Experiments 2 and 3 showed that: 1) people were attracted to an area of darkness when searching and avoided locations close to a window when hiding, 2) when search attempts were limited to three choices, people searched farther from origin and dispersed their locations more when hiding than when searching, and 3) informing people that they would need to recover their hidden objects altered their hiding behavior and increased recovery accuracy. Across all experiments, consistencies in location preferences emerged, with more preference for the middle of the room during hiding and more preference for corners of the room during searching. Even though the same people participated in both the hiding and searching tasks, it appears that people use different strategies to select hiding places than to search for objects hidden by others.

## Introduction

Humans and other species sometimes engage in behaviours designed to hide objects from others or to search for objects that are hidden in unknown locations. Effective hiding behaviour can protect valuable items from being pilfered, such as when we stash money in a secret location, or when a bird caches seeds for later use. Effective searching can both conserve time and energy and increase the chance of finding a valued object. Although hiding and searching strategies have been the focus of many investigations in non-humans (e.g. [1]–[3]), few studies have investigated the strategies people use. Understanding such strategies have many possible applications, such as the potential to inform law enforcement agents looking for hidden contraband or military personnel finding explosive devices. Our research investigates people’s hiding and searching strategies and aims to identify factors that influence where people hide and search for objects.

Studies of human adult search behaviors have generally focused on visual search for a target object among distractors in two-dimensional displays of artificial and natural scenes (e.g., [4]–[5]), or the concealment of objects within a visual display [6]. One recent study [7] investigated strategies used by people to search for a single object in a complex three-dimensional virtual maze. They reported that people searched systematically and preferentially followed the perimeter of the maze. A few studies have also investigated search strategies of children in real-space environments. Cornell and Heth [8] studied 6 to 8 year old children using a “treasure-hunt” type of task. They found that children generally avoided hiding objects near the entrance to the room and tended to cluster their choices. Older children showed more dispersion than younger children in selection of hiding locations. Wellman and colleagues [9] studied how preschool children (ages 3 to 5) searched for a missing item among eight possible hiding locations in a playground or room. They found that older children were more likely than younger children to search systematically among the hiding locations. Subsequent studies have also reported that children show more systematic (e.g., non-random, sequential) search patterns as they get older [10]–[12].

Our investigations of hiding and searching strategies in human adults use a navigation-based design modeled after the studies on animal food caching and recovery (for reviews, see [13]–[14]) and the aforementioned studies on children (e.g. [8]). In our initial work, adults were tested in a featureless, square room with nine possible hiding locations [15]. Participants hid and searched for three objects in a real or virtual room. In both environments, participants’ selection of locations differed from a uniformly random distribution and was different for hiding and searching. They selected locations farther from their starting location and dispersed their choices more when hiding than when searching. In addition, searching behavior was affected by prior experience hiding objects.

The present experiments extend our previous work [15] and address several additional questions about how people select locations when hiding or searching for objects. Across three experiments, we test five predictions.

### Hypothesis 1: Our Previous Findings [15] Will Generalize to More Complex Environments

To test this hypothesis we use larger, non-rectangular environments with over 70 cache locations. We expect to replicate our finding that in both real and virtual tasks, people show non-random location preferences that differ for hiding and searching. Although many studies have validated the use of virtual environments for investigations of spatial memory and navigation (see [16]–[17]), only the one previous study by Talbot et al. [15] has investigated whether people show similar hiding strategies in real and virtual spaces. Therefore, it seemed prudent to determine whether hiding and searching strategies remain similar within both spaces with a more complex room.

### Hypothesis 2: People will be Attracted to Locations in Dark Areas and Avoid Locations Near a Window when Hiding and Searching

Because the goal of hiding is to make objects difficult for others to find, we predict that people will be attracted to an area of darkness and will avoid areas in view of a window when hiding. If people search according to where they guess others will hide (i.e., use a ‘theory of mind strategy’, see [15]), the dark area and window may have the same attractive and repulsive effects on searching.

### Hypothesis 3: Limiting the Number of Search Attempts will Alter Searching Behavior

We expect that participants will search more strategically if they only have three tries to find all three objects. Thus, we expect that people will be less likely to search systematically and more likely to search selectively when their search attempts are limited. We expect this to reduce differences between hiding and searching.

### Hypothesis 4: Informing People that they must Later Recover their Hidden Objects will Influence their Hiding Behavior and Increase Recovery Accuracy

If people know that they must recover their objects, we expect that they will select locations based on a trade-off between two considerations: 1) how likely others will be to search the locations, and 2) how easily they can remember the locations. In contrast, uninformed participants may not consider the ease of remembering locations when making their hiding selections. We therefore expect to see a difference between informed and uninformed participants in the tiles chosen during hiding and a higher accuracy of recovery for the informed participants.

### Hypothesis 5: Certain Room Locations will be Consistently Preferred and Avoided

We predict that across all experiments, and despite changes in room features and procedures, consistencies will emerge in which locations are preferred and avoided. Similarities across experiments and conditions are expected to the extent that overall topological features play a role in location selections. Based on previous research [15], we expect that these locations will differ between hiding and searching.

## Methods

### Participants & Ethics Statement

The participants were University of Alberta undergraduate students. They received credit in their introductory Psychology class for participating. Written informed consent was obtained from all participants, and all procedures were approved by the University of Alberta’s Research Ethics Board. In Experiment 1, 102 participants (39 male, 63 female) with a mean age of 21 (range: 17–33) were tested in the real room and 141 participants (55 male, 81 female, 5 unreported) with a mean age of 19 (range: 17–42) were tested in the virtual room. Experiment 2 had 398 participants (164 male, 232 female, 2 unreported) with a mean age of 19 (range: 17–32). Experiment 3 had 394 participants (229 male, 153 female, 12 unreported) with a mean age of 19 (range: 17–45).

### Materials & Apparatus

#### Real room

**Figure 1:**
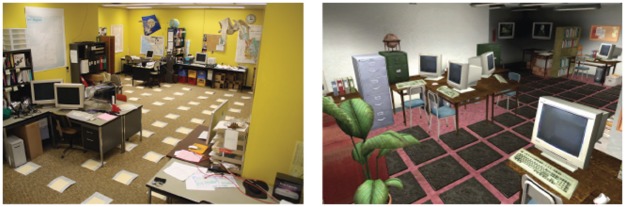
Screenshot of the real (left panel) and virtual (right panel) rooms used in Experiment 1.

The real room (Experiment 1 only) was a non-rectangular laboratory with 71 square laminate floor tiles. Tiles served as hiding and searching locations in all experiments (Figure 1, left). A file folder was velcroed to the top of each tile into which participants slid a paper card to indicate their selection. The room contained furniture (e.g., couches, tables, pictures), a dark corner to the left of the entry door, and a window to the outside in the corner opposite to the entry door.

#### Virtual room

The virtual room (Figure 1, right) was modeled after the real room and was created using the Hammer editor and Half-life 2 object libraries [18]. Virtual environments used the Source engine [19]. The virtual room had 73 clickable black squares that acted as tiles. In Experiment 1, the virtual room also contained furniture, a dark corner, and a window with a view of virtual characters moving and looking into the room. The locations of the dark corner and window were the same as in the real room. In Experiments 2 and 3, we removed the furniture to simplify the environment. For different groups, the room contained a window, a dark area or neither feature (empty room). In Experiment 2, the locations of the dark area and window were the same as in Experiment 1. In Experiment 3, the window and dark corner were both located in the corner directly in front of the room entrance. The room was viewed from a first-person perspective with a player height of 183 cm.

### Procedure

In all experiments, participants were tested in both a hiding task, in which they hid objects under the floor tiles, and a searching task, in which they searched under floor tiles to find hidden objects. Order of exposure to the tasks was counterbalanced across participants and assignment to groups was randomized. In the hiding task, participants were told that their goal was to hide three objects under tiles so that they would be difficult to find by another person. In the searching task, participants were instructed to select tiles that were most likely to contain an object hidden by someone else. Experiment 3 also included a recovery task in which participants had three attempts to find their previously hidden objects. The recovery task was presented after participants completed both hiding and searching tasks.

#### Real room

In the hiding task, participants hid three index cards numbered 1 to 3 in file folders on top of floor tiles, placing at most one card per folder. For the searching task, participants were given a stack of numbered “searching” cards (that differed in color from the hiding cards) and were told to search for three cards hidden by someone else and to slide a card into each location they checked. For both tasks, one researcher stood still on the right side of the door while a second researcher stood by the window and recorded all tile selections. These recordings were confirmed after the trial by the card locations. There was no time limit placed on the participants in either task.

#### Virtual task

Participants started with tutorials that provided experience in navigating the virtual environment by walking through a series of corridors, as well as practice hiding and searching in empty rooms. Participants were instructed that to select a tile, they needed to be close (within 183 cm), point to it with the cursor, and then click on it.

After the tutorials, participants proceeded to the experimental hiding and searching tasks. These tasks were conducted in a different room than the tutorials. In both hiding and searching, the participant started at the entrance to the room (point of origin). In Experiments 1 and 2, instructions were overlaid on the screen for nine seconds, during which participants could move within the room but could not click on the tiles. In Experiment 3, the instructions were presented on a black screen before entering the room. A one-second delay followed each tile selection before another tile could be selected.

In the hiding tasks, participants were told that they had three objects to hide. The task ended when all three objects were hidden or after a maximum of 120 seconds. For each valid click, a message indicated that they had hidden an item and how many items remained to be hidden. In addition, a light appeared over the selected tile for five seconds. Participants could only hide one object per tile. Repeated choices of a tile produced an error message.

In the searching task, participants searched for three hidden items. In Experiments 1 and 2, a counter was continuously displayed that started at 100 points, decreased by 1 point for each empty tile selected and increased by 15 points for each object found. This was designed to provide motivation for searching efficiently. Clicking on a tile produced a message indicating whether or not an object was found and how many objects remained. If an object was found, a five second light appeared above the tile. The searching task ended when all objects were found or after a maximum of 120 seconds. In Experiment 3, search attempts were limited to three choices and there was no counter. A light appeared above each selected tile but there was no feedback about whether an object was found. The task ended after the third choice.

In the recovery task (Experiment 3 only), participants were given three choices to find their previously hidden objects. A light appeared above each selected tile but there was no feedback regarding the accuracy of their selections. Participants were randomly assigned to “informed” or “uninformed” subgroups. Informed participants were told in the tutorial and immediately before hiding that they would need to later recover their hidden objects. The upcoming recovery task was not mentioned to uninformed participants.

Following each task, participants clicked on the door to exit the room. After completing all tasks, participants were retested in a different room for the purposes of another experiment, which is reported elsewhere [20].

### Data Analysis

#### Metric measures

We computed two metric measures for each participant’s searching and hiding choices. The first measure, distance from origin, was calculated as the Euclidean distance from the starting position of the participant to the center of the first tile selected. The second measure, perimeter, was calculated by summing the Euclidian distance from the first tile to the second tile, the second tile to the third tile, and the third tile to the first tile (ignoring walls; the center of a tile was always used for these calculations).

All metric measures were analyzed using repeated measures ANOVAs, with Task (hiding; searching) as the repeated factor. Order (HS: hiding then searching; SH: searching then hiding) and Gender (male; female) were between-subjects factors. Data were collapsed across Order and Gender for subsequent analyses when these factors were not significant. In Experiments 2 and 3, room configuration (Dark, Window, Empty) was included as a between-subjects factor. We report the means (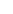) and standard error of the mean (SEM) for all statistically significant results (p<.05) when analyzing metric measures (distance from origin; perimeter) in Tables S1 and S2. All post-hoc comparisons were Bonferroni corrected. Cohen’s d effect sizes were computed using G*Power [21].

#### Analysis of choice frequencies

For choice frequency analyses, we used only the first bin choice because later choices in searching could be contaminated by whether an object was or was not found. In order to provide sufficiently high choice frequencies per location for non-parametric analysis, we pooled participants’ first hide and search choices into three bins. Bins were designed to distinguish between choices that fell in the corners and edges of the search space, choices that fell in the middle of the search space, and choices that fell between the middle and edges. To create these bins we first represented all tiles on a grid similar to those displayed at the bottom of Figure 3. For each tile we then 1) counted the number of grid locations that intervened between the tile and the edge of the grid space separately for each cardinal direction (N, E, S, W), using a count of zero for tiles immediately adjacent to the edge of the grid space in a given direction, 2) found the vertical (V) and horizontal (H) minima using: V = min(N,S) and H = min(W,E), 3) computed an average distance (D) for each tile using: D = average (sqrt(H), sqrt(V)). As a result, each tile was labeled with a single scalar, D, which was used to partition all tiles into three bins. Binning was accomplished by computing the range of D over all tiles [min(D),max(D)], and then dividing the range into three parts. Because several tiles had the same D value, the number of tiles in each bin was not completely equal. The expected frequency of choices to a bin (based on a uniform distribution) was derived by dividing the number of tiles in a bin by the total number of tiles in the room. Frequency data were then analyzed using Chi square tests for goodness of fit. To determine if choices were non-random, we compared observed frequencies to frequencies expected on the basis of random sampling with a uniform distribution. To determine if searching choices differed from hiding choices, we compared the observed bin frequencies when searching to the expected frequencies based on the hiding distribution. For Experiments 2 and 3, choice frequencies were collapsed across room configuration conditions for these analyses.

#### Environmental feature analysis

To examine the effect of darkness on participants’ hiding and searching behaviour, tiles were separated into two bins according to whether they fell in the dark area (Experiment 2: dark tiles = 3, other tiles = 70; Experiment 3: dark tiles = 4, other tiles = 69). The dark area was determined by evaluating the brightness of each tile. A tile was considered in the dark area if its brightness value was less than one standard deviation from the average brightness of all tiles (brightness is an object property in the game-editor we used; the brightness of an object changed depending on the placement and intensity of light sources in the environment). To examine the effect of the window, tiles were separated into two bins according to whether they fell within an area near the window The area was an equilateral triangle with the apex at the center of the window and each side measuring 3.66 m. To be considered a window tile, at least 50% of the tile had to fall within this triangular area. (Experiment 2: window tiles = 7, other tiles = 66; Experiment 3: window tiles = 12, other tiles = 61).

We separated tiles into the same bins for the empty condition to serve as a comparison baseline for both the dark and window conditions. We used Chi-square tests to compare the frequency of first choices in the dark or window condition to the empty condition for both hiding and searching. If a difference between the empty and the room feature (dark or window) condition was found, additional analyses of the bin choices for the feature condition were conducted.

#### Recovery analysis (Experiment 3 only)

To determine if informed participants were more successful than uninformed participants in recovering their hidden objects, we examined the accuracy of participants’ first choice on recovery as well as how many correct locations they selected on their three choices. These were analyzed with Chi-square tests.

## Results

### Experiment 1

Experiment 1 addressed Hypothesis 1 using both real and virtual environments.

#### Results

**Figure 2:**
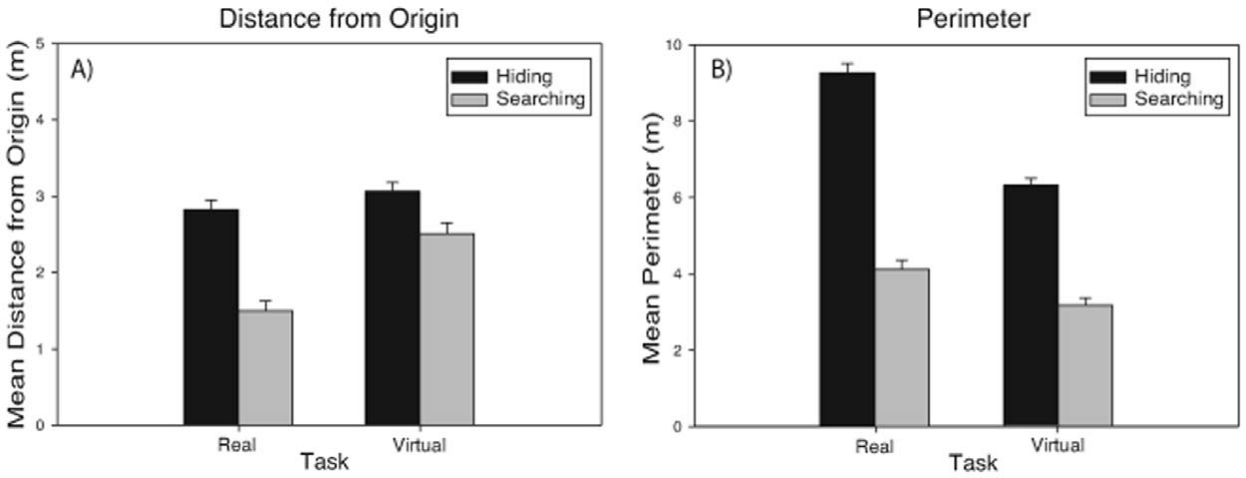
Mean distance from origin (A) and mean perimeter (B) of participant’s choices when hiding (black bars) and searching (grey bars) in both the real and virtual rooms. All distances are in meters.

***Distance from origin.*** In both the real and virtual rooms, participants traveled farther from the start location when hiding than when searching. Analyses confirmed that distance from origin was greater for hiding than for searching in both the real [F(1,97) = 66.89, p<.001 η_p_^2^ = .38] and virtual [F(1,139) = 9.75, p<.01, η_p_^2^ = .07] rooms (see Figure 2, left panel; see Table S1 for means and SEMs). There were no significant main effects of Order or Gender in either room [p>.05], and no significant Order x Task or Gender x Task interactions in the virtual room. However, significant Order x Task [F(1, 97) = 6.31, p<.05, η_p_^2^ = .06] and Gender x Task [F(1,97) = 4.85, p<.05, η_p_^2^ = .05] interactions were observed in the real room (See Table S2 for means and SEMs).

*Post-hoc* tests (Bonferroni corrected to α = .0125) on the significant Order x Task interaction observed in the real room revealed that regardless of Order, participants traveled significantly farther from origin when hiding than when searching [HS: t(1,49) = 4.00, p<.001, d = .66; SH: t(1,51) = 6.74, p<.001, d = 1.48]. Additionally, when hiding, participants who searched first (SH) traveled significantly farther than participants who hid first (HS), [t(1,100) = 3.05, p<.01, d = .60]. There was no significant effect of Order on distance from origin when searching [p>.05].

*Post-hoc* tests (Bonferroni corrected to α = .0125) on the significant Gender x Task interaction observed in the real room revealed that both males and females traveled further from origin when hiding than when searching [males: t(1,38) = 6.17, p>.001, d = .99; females: t(1,61) = 4.75, p>.001, d = .60]. However, there was no significant effect of gender on distance from origin when hiding or searching [p<.0125].

***Perimeter.*** Participants clustered their choices more (had a smaller perimeter) when searching than when hiding in both the real [F(1,100) = 200.2, p<.001, η_p_^2^ = 0.67] and virtual [F(1,139) = 167.77, p<.001, η_p_^2^ = 0.55] rooms (see Figure 2, right panel; see Table S1 for means and SEMs). No other main effects or interactions were significant [p>.05].

#### Choice frequencies

**Figure 3:**
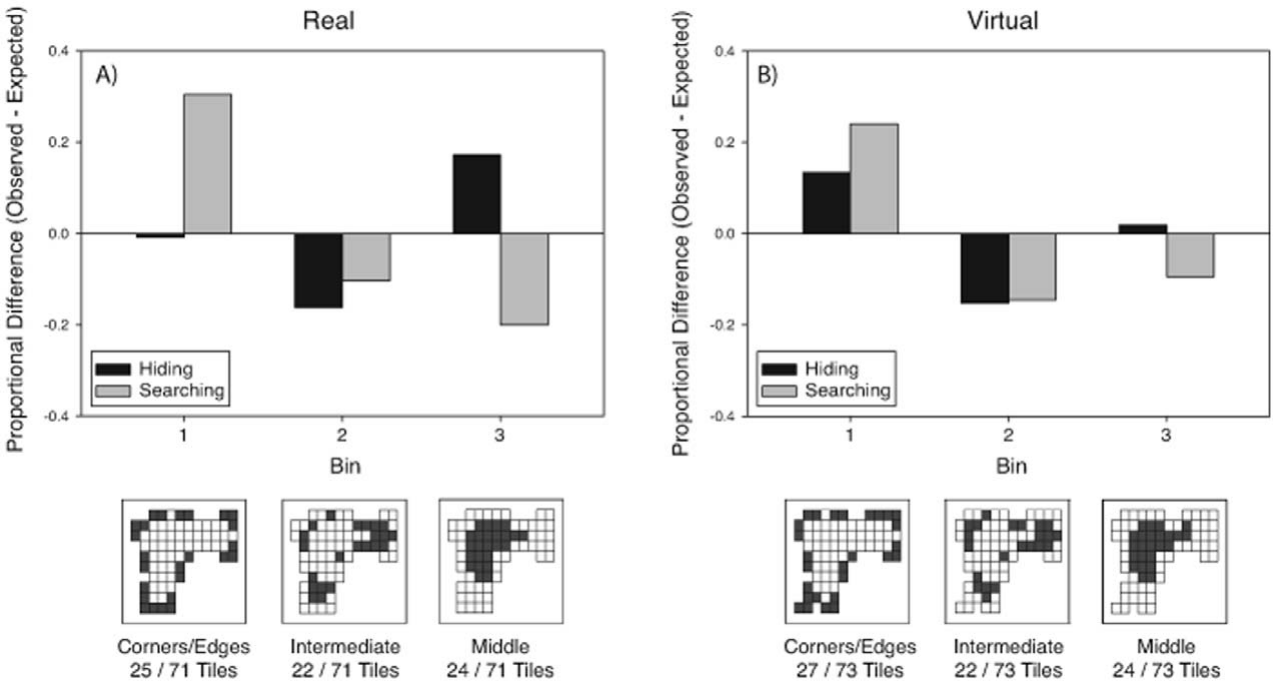
Proportional difference scores for each bin when hiding (black bars) and searching (grey bars) in the real (A) and virtual (B) rooms in Experiment 1. Proportional difference scores were calculated by subtracting the proportion of choices observed from the proportion of choices expected given a uniform distribution. The bottom images are schematics of the tile layouts in each room. Each square denotes a tile, and darkened squares indicate the tiles that fell within a given bin.

**Figure 4:**
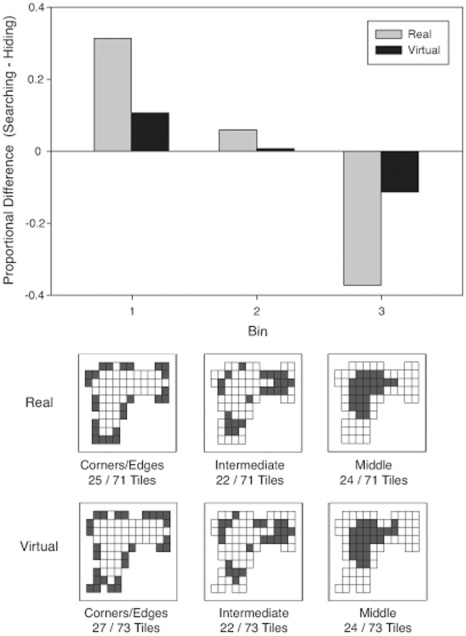
Proportional difference scores for choices made when searching and hiding. Scores were calculated by subtracting the proportion of choices made to each bin when searching from the portion of choices made to each bin when hiding. All proportions were normalized to the number of tiles in each bin. The bottom images are schematics of the tile layouts in each room. Each square denotes a tile, and darkened squares indicate the tiles that fell within a given bin.

***Real room.*** There was no significant effect of Order on bin choice during hiding or searching, [p>.05]. As shown in left panel of Figure 3, frequencies of binned tile choices differed from a uniform distribution for both tasks [Hiding: 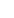
(2, *N* = 102) = 17.39, p<.001, Φ_c_ = .29; Searching 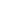
(2, *N*  = 102) = 43.34, p<.001, Φ_c_ = .46]. During both tasks, people chose locations in intermediate locations (Bin 2) less frequently than expected based on a uniform random distribution. However, the pattern of choices for Bins 1 (corner and edges) and 3 (middle) differed between hiding and searching. The bins chosen for searching differed from the frequency expected based on the hiding distribution, [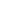
(2, *N* = 102) = 59.43, p<.0001, Φ_c_ = .54, see Figure 4]. Participants were more likely to choose locations near the corners and edges (Bin 1) and to avoid locations in the middle (Bin 3) when searching than when hiding.

***Virtual-room.*** There was no significant effect of Order on bin choice during hiding or searching, [p>.05]. As shown in the right panel of Figure 3, frequencies differed from a uniform distribution in both tasks [Hiding: 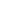
(2, *N* = 141) = 17.65, p<.001, Φ_c_ = .25; Searching: 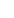
(2, *N* = 141) = 35.61, p<.001, Φ_c_ = .36]. During both tasks, people chose locations close to the corner and edges (Bin 1) more frequently, and choices in intermediate locations (Bin 2) less frequently than expected based on a uniform random distribution. Furthermore, the bins chosen during searching differed from the expected distribution based on the bins chosen during hiding, [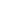
(2, *N* = 141) = 8.44, p<.05, Φ_c_ = .17 see Figure 4]. As in the real room, participants chose locations near the corners and edges (Bin 1) and avoided locations in the middle (Bin 3) more when searching than when hiding.

### Experiment 2

Experiment 2 was designed to extend the findings of Experiment 1 and to test Hypothesis 2.

#### Results

**Figure 5:**
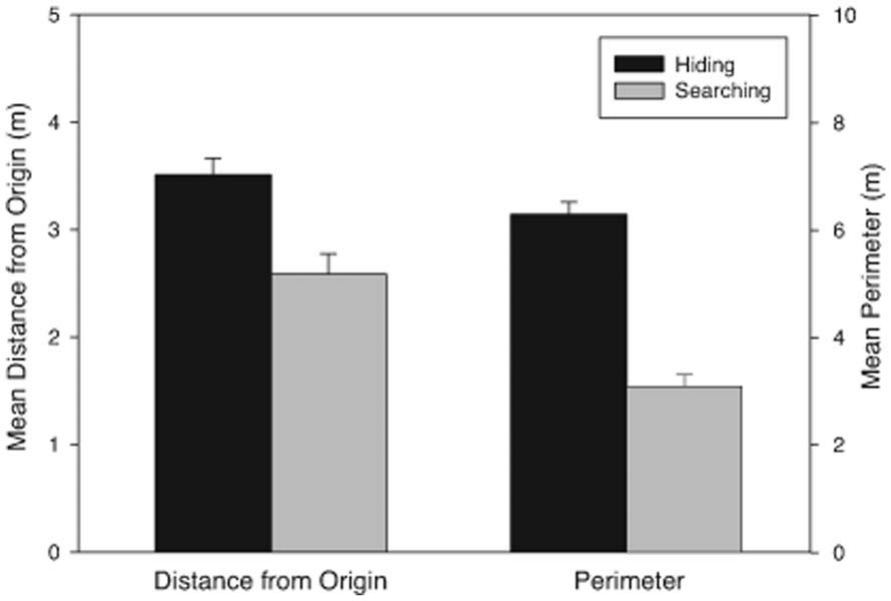
Mean distance from origin (left bars) and mean perimeter (right bars) traveled by participants when hiding (black bars) and searching (grey bars) in Experiment 2. All distances are in meters.

***Distance from origin.*** As in Experiment 1, participants traveled farther from origin when hiding than when searching, [F(1, 392) = 27.43, p<.001, η_p_^2^ = 0.07] (see Figure 5, see Table S1 for means and SEMs). No other effects were significant, [*p*>.05].

***Perimeter.*** As in Experiment 1, participants clustered their choices more when searching than when hiding, [F(1, 392) = 627.08, p<.001, η_p_^2^ = 0.62] (see Figure 5, see Table S1 for means and SEMs). There were no other significant effects, [*p*>.05].

### Choice Frequencies

There was a significant effect of Order on bin choice during hiding [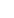
(2, *N* = 398) = 6.71, p<.05, Φ_c_ = .09]. Specifically, participants who hid first (HS) preferred Bin 1 (corner and edges), whereas those who searched before they hid (SH) preferred Bin 3 (middle). There was no significant effect of Order on binned choices during searching [p>.05]. For the remaining tests, we collapsed across Order.

**Figure 6:**
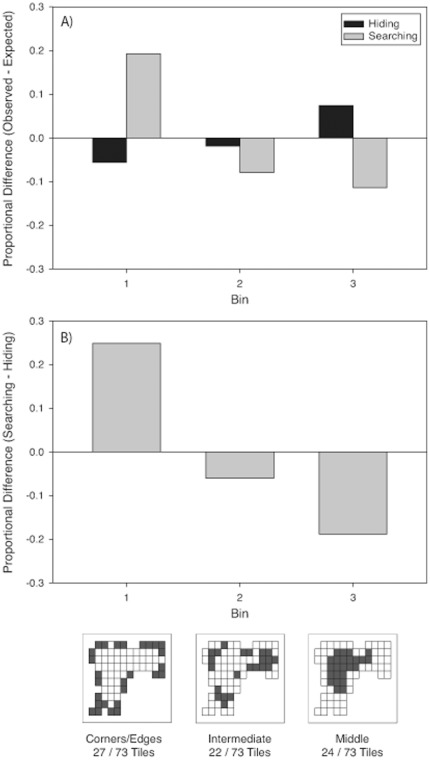
Proportional difference scores for hiding and searching in Experiment 2. (*A*) Proportional difference scores for hiding (*black bars*) and searching (*grey bars*) in each bin in Experiment 2. Proportional difference scores were calculated by subtracting the proportion of choices observed from the proportion of choices expected given a uniform distribution. (*B*) Proportional difference scores for choices made when searching and hiding. Scores were calculated by subtracting the proportion of choices made to each bin when searching from the portion of choices made to each bin when hiding. All proportions were normalized to the number of tiles in each bin. The bottom images are schematics of the tile layouts in the room. Each square denotes a tile, and darkened squares indicate the tiles that fell within a given bin.

Participants’ choices were non-random in both tasks, [Hiding: 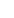
(2, N = 398) = 10.52, p<.01, Φ_c_ = .12; Searching: 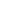
(2, N = 398) = 63.9, p<.0001, Φ_c_ = .28], and the frequency of bin choices during searching differed from the expected frequency based on the hiding distribution [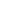
(2, N = 398) = 118.49, p<.001, Φ_c_ = .39] (see Figure 6). Participants were more likely to choose tiles in Bin 1 (corner and edges) and less likely to choose tiles in Bin 3 (middle) when searching than when hiding.

#### The role of environmental features

**Figure 7:**
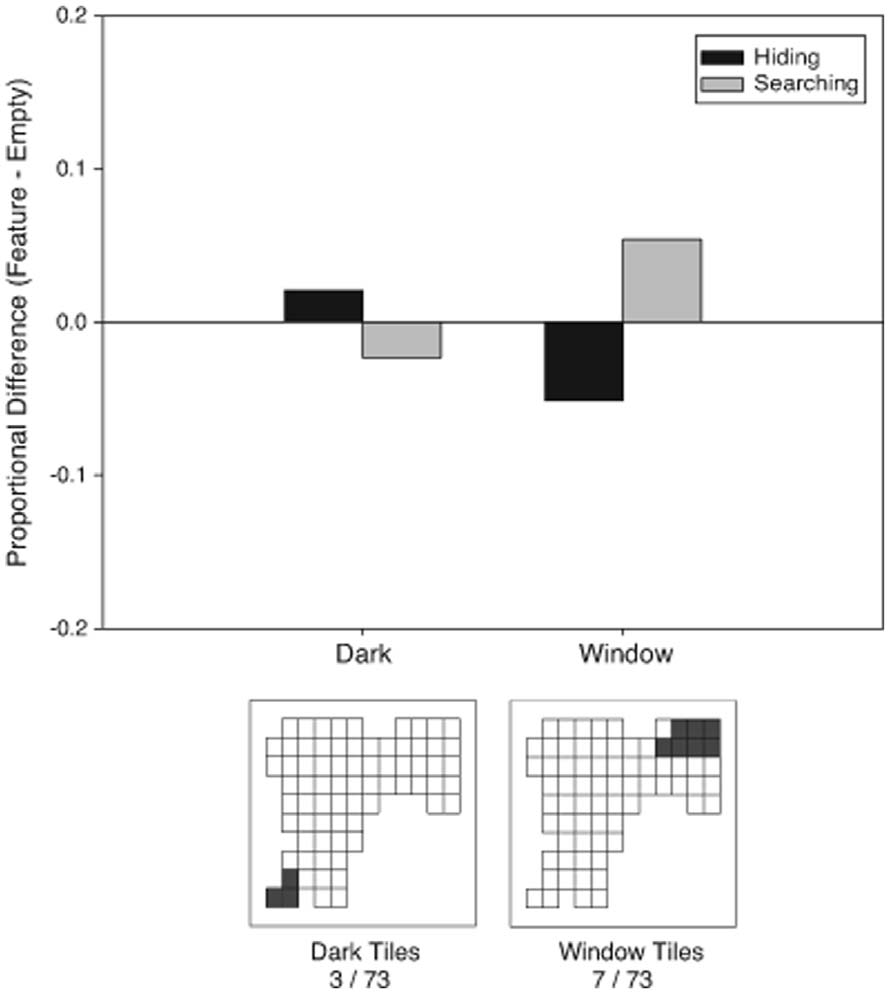
Proportional difference scores for the dark (left bar pair) and window (right bar pair) areas for hiding (black bars) and searching (grey bars) in Experiment 2. Scores were calculated by subtracting the proportion of choices to the tiles of interest from the proportion of choices to the same tiles in the empty room. The bottom images are schematics of the tile layouts in the room. Each square denotes a tile, and darkened squares indicate the tiles of interest used for comparison to the empty room.

***Darkness.*** The frequency of first choices of tiles in the dark corner was not different from the frequency of first choices of the same tiles in the empty condition for hiding or searching, [p>.05]. Thus, darkness had no significant effect on first tile choice (Figure 7).

***Window.*** When hiding, participants chose the window tiles significantly less often in the window condition than in the empty condition, [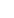
(1, N = 128) = 4.51, p<.05, Φ = .19] (Figure 7). Thus, the window had a repulsive effect when hiding. There was no significant effect of the window when searching, [p>.05].

Additionally, in the window condition, participants chose window tiles significantly more when searching than expected based on their hiding distributions [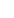
(1, N = 135) = 2.84, p<.01, Φ = .15]. Choice of these tiles did not differ between hiding and searching in the empty condition [p>.05].

### Experiment 3

Experiment 3 further tested Hypothesis 2 and tested Hypotheses 3 and 4.

#### Results

**Figure 8:**
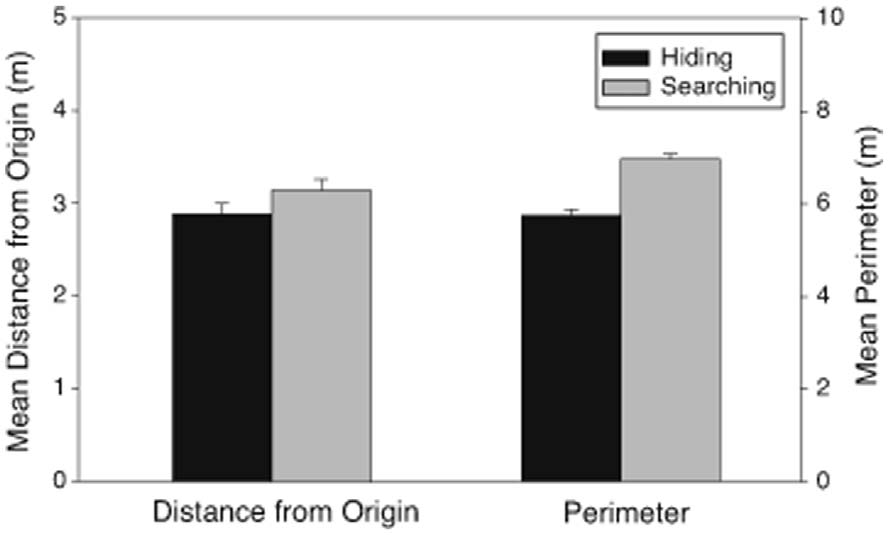
Mean distance from origin (left bar pair) and mean perimeter (right bar pair) traveled by participants when hiding (black bars) and searching (grey bars) in Experiment 3. All distances are in meters.

***Distance from origin.*** Unlike in Experiments 1 and 2, participants travelled farther from origin when searching than when hiding [F(1, 388) = 7.08, p<.01, η_p_^2^ = .02] (see Figure 8; see Table S1 for means and SEMs). There was also a significant main effect of Order, in which participants traveled farther from origin if they hid prior to searching (HS, 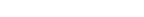) than if they searched prior to hiding (SH, 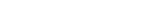 [F(1, 388) = 4.29, p<.05, η_p_^2^ = .01] and a significant Task x Order interaction, [F(1,388) = 8.08, p<.01, η_p_^2^ = .02] (see Table S2 for means and SEMs). No other effects were significant, [p>.05].

Post-hoc tests (Bonferroni corrected to α = .0125) on the significant Task x Order interaction revealed that participants in group SH traveled significantly farther from origin when searching than when hiding, [t(1,200) = −3.94, p<.001, d = .26]. For participants in group HS, distance from origin was not significantly different when searching than when hiding, [p>.05]. When hiding, distance from origin was significantly higher for group HS than for group SH, [t(1,392) = 3.55, p<.001, d = .35]. There was no similar effect when searching, [p>.05].

***Perimeter.*** Also contrary to Experiments 1 and 2, participants clustered their choices more when hiding than when searching, [F(1, 388) = 56.63, p<.001, η_p_^2^ = .13] (see Figure 8; see Table S1 for means and SEMs). No other effects were significant, [p>.05].

#### Choice frequencies

**Figure 9:**
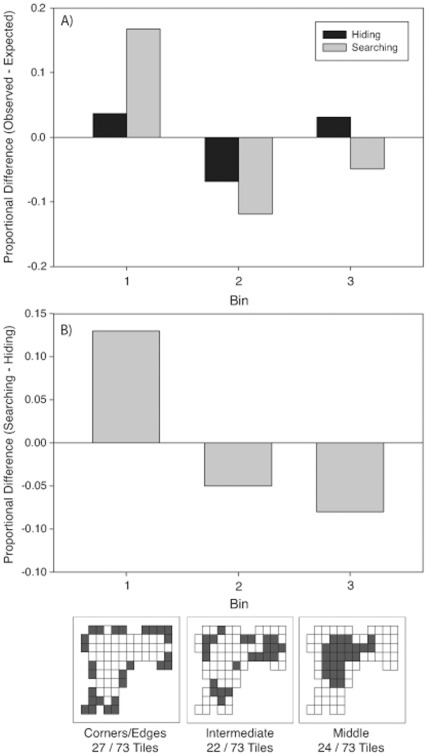
Proportional difference scores for hiding and searching in Experiment 2. (*A*) Proportional difference scores for hiding (*black bars*) and searching (*grey bars*) in each bin in Experiment 3. Proportional difference scores were calculated by subtracting the proportion of choices observed from the proportion of choices expected given a uniform distribution. (*B*) Proportional difference scores for choices made when searching and hiding. Scores were calculated by subtracting the proportion of choices made to each bin when searching from the proportion of choices made to each bin when hiding. All proportions were normalized to the number of tiles in each bin. The bottom images are schematics of the tile layouts in the room. Each square denotes a tile, and darkened squares indicate the tiles that fell within a given bin.

There was no significant effect of Order on bin choice during hiding or searching, [p>.05]. Participant’ choices were non-random in both tasks, [Hiding: 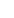
(2, N = 394) = 8.95, p<.05, Φ_c_ = .11; Searching: 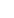
(2, N = 394) = 52.45, p<.0001, Φ_c_ = .26] and bin choices during searching differed from the expected frequencies based on their hiding distribution [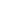
(2, N = 394) = 28.43, p<.001, Φ_c_ = .19] (see Figure 9). As in both previous experiments, participants were more likely to choose tiles in Bin 1 (corner and edges) and less likely to choose tiles in Bin 3 (middle) when searching than when hiding.

#### The role of environmental features

**Figure 10:**
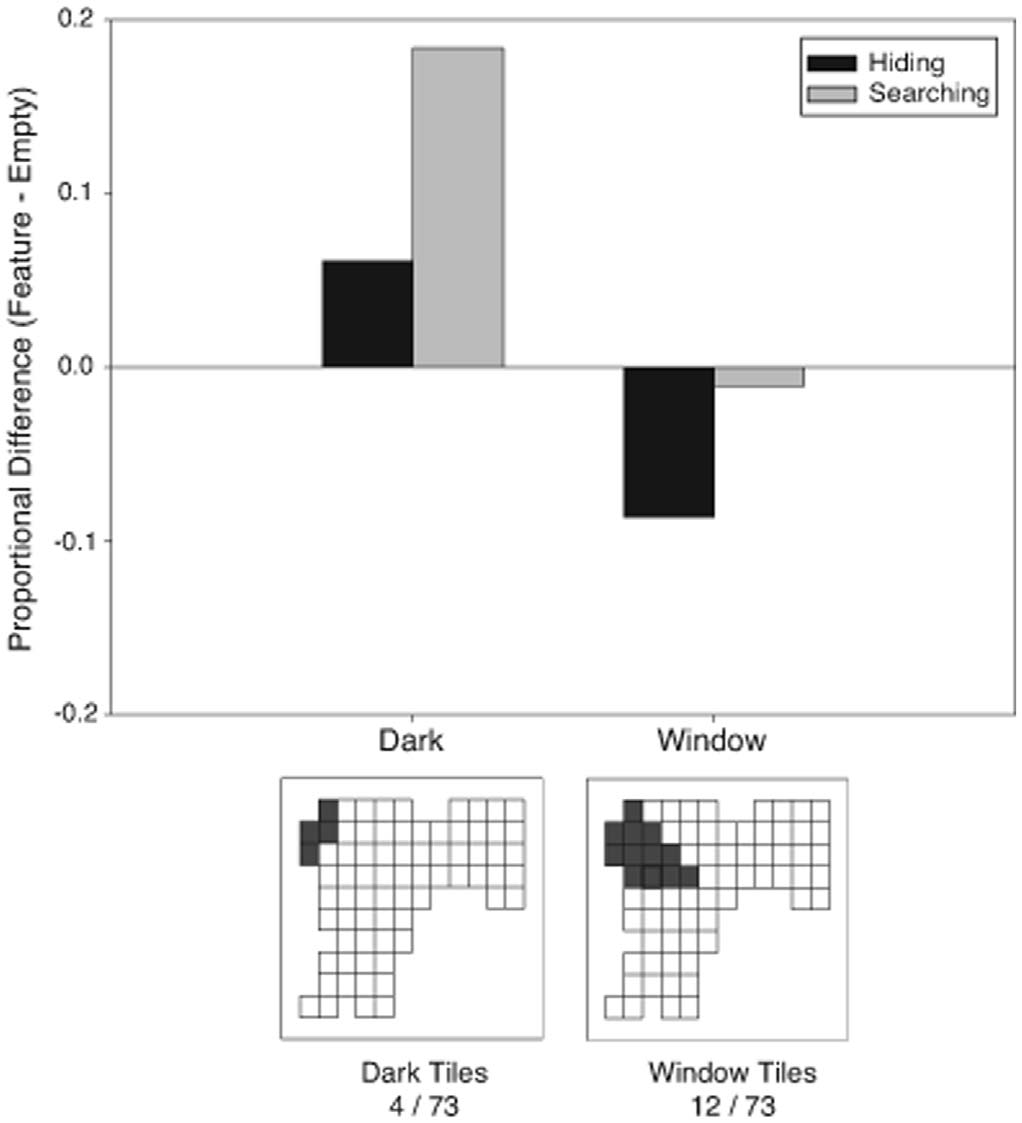
Proportional difference scores for the dark (left bar pair) and window (right bar pair) areas for hiding (black bars) and searching (grey bars) in Experiment 3. Scores were calculated by subtracting the proportion of choices to the tiles of interest from the proportion of choices to the same tiles in the empty room. The bottom images are schematics of the tile layouts in the room. Each square denotes a tile, and darkened squares indicate the tiles of interest used for comparison to the empty room.

***Darkness.*** Figure 10 shows the frequency of first choices to dark tiles when hiding and searching in the dark and empty conditions. There was no significant difference between the dark and empty condition when hiding, but when searching, participants significantly chose these tiles more in the dark condition than the empty condition, [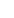
(1, *N* = 260) = 13.63, p<.001, Φ = .23].

Additionally, in the dark condition, participants chose the dark tiles significantly more when searching than expected based on their distribution of choices when hiding, [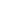
(1, N = 130)  = 39.87, p<.001, Φ = .55]. This finding suggests that participants were more attracted to locations partially obscured by darkness when searching than when hiding. Although participants in the empty condition also chose these tiles more when searching compared to their distribution during hiding, [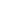
(1, N = 129) = 7.4, p<.01, Φ = .24], the effect was much weaker.

***Window.*** As shown in Figure 10, when hiding, participants chose the window tiles significantly less in the window condition than in the empty condition, [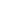
(1, N = 129) = 6.34, p<.05, Φ = .22]. When searching, there was no difference between the window and empty conditions in the frequency of choices to the window tiles, [p>.05]. The distribution of tile choices during searching did not differ from that expected based on the hiding distribution in either the window or the empty condition, [p>.05]. Thus, the presence of a window had a repulsive effect on participants’ hiding behaviour, but had no effect on participant’s searching behaviour.

#### The role of being informed

**Figure 11:**
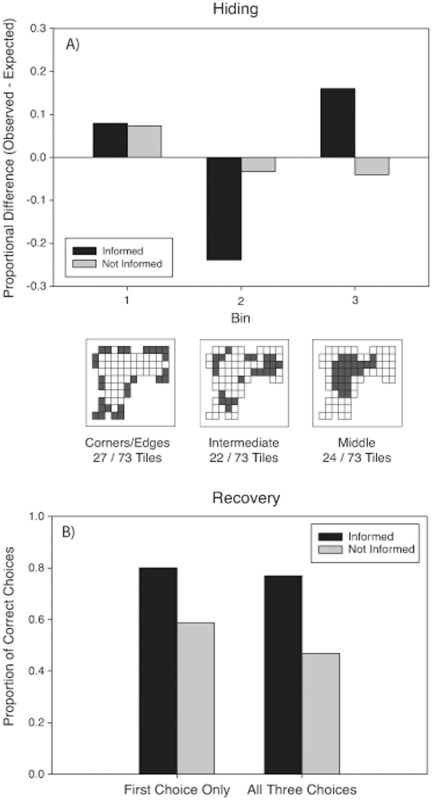
Proportional difference scores for hiding and searching in Experiment 2. (*A*) Proportional difference scores for informed (*black bars*) and uninformed (*grey bars*) participants in each bin when hiding in Experiment 3. Proportional difference scores are calculated by subtracting the proportion of choices expected given a uniform distribution from the actual proportion of choices made to each bin. (*B*) Proportion of location choices made to locations chosen when hiding on participants’ first choice and all three choices in the recovery task. Proportion of correct choices are separated by whether participants were informed (*black bars*) or uninformed (*grey bars*).

Informed and uniformed participants did not differ significantly in distance from origin or perimeter [p>.05]. However, the two groups differed in their bin choice frequencies when hiding [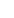
(2, N = 394) = 7.03, p<.05, Φ_c_ = .10] (Figure 11a). Specifically, informed participants were more likely to hide in Bin 3 (center) and less likely to hide in Bin 2 (intermediate) than uninformed participants.

Recovery of a previous hiding location was significantly higher for informed participants than for uniformed participants on their first choice [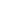
(1, N = 394) = 21.25, p<.0001, Φ = .23] and for all three choices [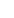
(1, N = 182) = 113.37, p<.0001, Φ = .54] (Figure 11b).

### Consistency of Location Preferences across Experiments

**Figure 12:**
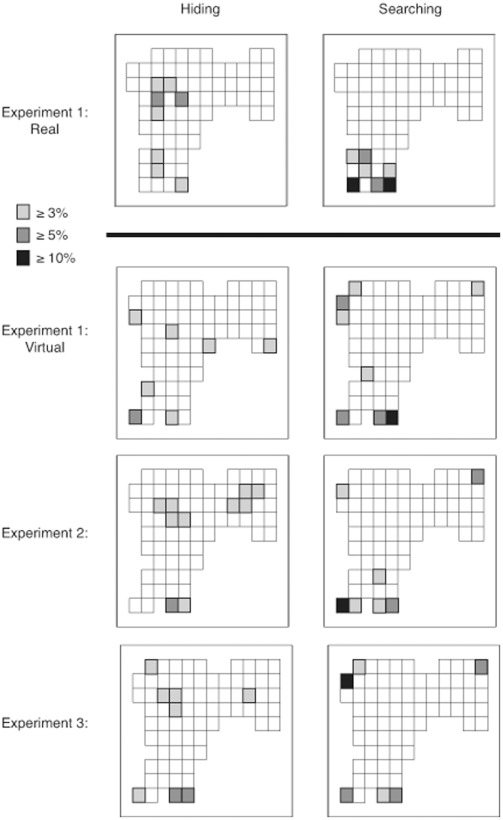
Figure showing individual tiles chosen by participants on their first choice when hiding (left plots) and searching (right plots) in each experiment. The shade of grey scale indicates the percentage of first choices that participants made to a given bin.

**Figure 13:**
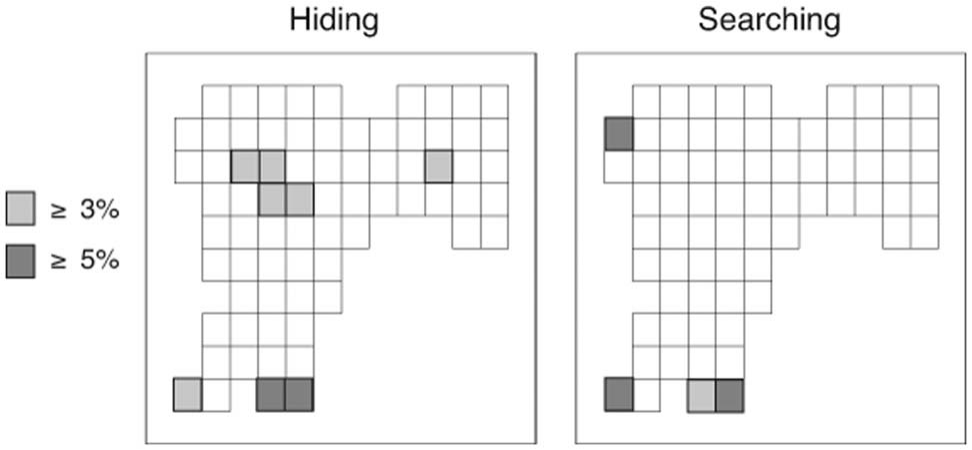
Figure showing individual tiles chosen by participants on their first choice when hiding (left plot) and searching (right plot) when pooled across all virtual tasks. The shade of grey scale indicates the percentage of first choices that participants made to a given bin.

To test Hypothesis 5, we calculated which tiles were chosen by more than 10%, 5% and 3% of participants in both hiding and searching tasks for each experiment (see Figure 12). Additionally, we summed the frequencies of first choices to each tile for all three virtual environments for both hiding and searching and highlighted the tiles that contained more than 5% and 3% of the choices (see Figure 13). Preferred hiding locations tended to be in the center of the search space, whereas preferred searching locations were primarily in the entrance and the corners.

## Discussion

Our experiments were designed to enhance understanding of adult hiding and searching behaviour. Discussion of our results is organized according to our hypotheses.

### Hypothesis 1: Previous Findings will Generalize to More Complex Environments

Three main results reported in Talbot et al. [15] replicated in our larger, more complex environments. First, the locations participants selected when hiding and searching differed from a uniform random distribution. Second, Experiment 1 found that in both real and virtual environments, people were more likely to choose locations near the corners and edges (Bin 1) and to avoid locations in the middle (Bin 3) when searching than when hiding. This similar pattern for real and virtual spaces supports previous evidence that virtual environments provide a good model for investigating spatial strategies (e.g., [15], [17]). Third, in both Experiments 1 and 2, participants traveled farther from their starting location and clustered their first three choices more when hiding than when searching. However, we did not replicate the finding that prior experience hiding altered search behavior.

### Hypothesis 2: People will be Attracted to Locations in Dark Areas and Avoid Locations Near a Window when Hiding and Searching

Although the area of darkness had no significant effect on hiding or searching in Experiment 2, it did have the predicted attractive effect on searching in Experiment 3. The different location of the dark area could account for the difference in results between the two experiments. Specifically, the dark area might have had less of an attractive effect in Experiment 2 because it was near the entrance to the room. The window had the predicted repulsive effect on hiding in both experiments, but it had no significant effect on searching behavior in either experiment. Thus people seem to avoid hiding in front of a window, but this feature does not discourage searching.

### Hypothesis 3: Limiting the Number of Search Attempts will Alter Searching Behavior

In Experiment 3, which limited searching to three choices, the perimeter and distance from origin measures showed differences between hiding and searching that were opposite to those found in Experiments 1 and 2. Specifically, participants in Experiment 3 traveled further from origin and dispersed their choices more when searching than when hiding. The difference between the experiments in these measures appeared to be driven primarily by increased origin and perimeter values during searching; the metrics were quite similar across experiments for hiding. The change in searching behavior is consistent with our prediction that people would be less likely to choose systematically (for example by starting at the entrance and selecting adjacent locations) and more likely to choose selectively when search choices were limited. Nevertheless, the pattern of location choices was similar across the three experiments. Specifically, in all experiments, participants were more likely to choose a location in the middle of the search space, and less likely to choose a location near the corner or edges of the room when hiding than when searching. Thus, limiting the allowed number of searches increased the distance from origin of the first choice and the perimeter of three choices, but it did not influence preference for particular topographical features of the search space. Specifically, a perusal of Figure 12 shows that during searching, participants in all experiments showed an affinity for the corners. Searching in Experiment 3, however, differed from the other experiments in that the highest density was shifted to a corner away from the point of origin.

### Hypothesis 4: Informing people that they must Later Recover their Hidden Objects will Influence their Hiding Behavior and Enhance Recovery Accuracy

The results of Experiment 3 revealed that informing participants about the recovery task had no effect on the distance from origin or perimeter measures during hiding. However, informed participants were more likely than uninformed participants to avoid the intermediate room locations (Bin 2) and favour the middle locations of the room (Bin 3). In support of our hypothesis, informed participants also showed higher recovery accuracy on their first choice and they recovered more of their hiding locations within three choices than uninformed participants.

### Hypothesis 5: Certain Room Locations will be Consistently Preferred and Avoided

Task-specific location preferences appeared in all three experiments. Specifically, when searching, participants frequently chose tiles that were near the entrance to the room and in the corners and rarely chose tiles in the center of the space. When hiding, participants tended to select tiles that were near entrance as well as tiles at the center of the search space. Combined across experiments, we see that people do not just hide where they search, or search where they hide. Instead they prefer different locations when hiding than when searching. Perhaps one of the most interesting implications of these results is that when searching for tiles hidden by others, people may apply a theory of mind and “overthink” where others might hide objects. For example, attraction to the less visible tiles in a dark area was seen for searching behavior but not for hiding behavior. When searching, people frequently looked in the corner tiles but did not often search in the high visibility middle areas of the room, which is where people often hid their objects. It is interesting that these differences emerged given that the same people participated in both the hiding and searching tasks.

### Conclusions and Future Directions

This research showed that even in a complex space with a large set of hiding locations, people show systematic location preferences that differ for hiding and searching. Moreover similar patterns of results appeared in virtual and real environments. We also showed an effect of two room features, a window and an area of darkness, on hiding and searching, respectively. Undoubtedly, other environmental features (e.g., isovists and isovist fields [22]) are likely to play a role in different environments or scales of space (e.g. geographical space [23]). Our results suggest that virtual environments may provide a practical means of identifying important environmental features and comparing strategies across different scales of space. Finally, we showed an effect of two procedural factors. Specifically, limiting the number of search attempts influenced metric measures of searching and informing people that they would need to recover their hidden objects influenced their hiding location preferences and recall ability. Taken together, these findings encourage the continued use of virtual navigation tasks to further investigate hiding and searching strategies in people, for example by exploring the strategies people use in larger multi-room or outdoor environments, or with different types of hiding places (such as drawers or bookcases). Furthermore, our paradigm has proven useful for developing models of human behavior for use in game development and the creation of realistic artificial agents [20]. Future work may use this approach to more directly test applications for both military and police activities such as improvised explosive device (IED) detection and contraband search.
